# Utility of Ultrasound-Based Dynamic Assessment in Physical Therapy for Far-Lateral Lumbar Disc Herniation: A Case Report

**DOI:** 10.7759/cureus.105249

**Published:** 2026-03-15

**Authors:** Hiromichi Mizuno, Masashi Kawabata, Ryosuke Furuta, Hiroki Nakagawa, Kenichiro Tanaka

**Affiliations:** 1 Department of Rehabilitation, Tanaken Spine and Ophthalmology Clinic, Ichinomiya, JPN; 2 Department of Rehabilitation, Kitasato University School of Allied Health Sciences, Sagamihara, JPN; 3 Department of Rehabilitation, Nagoya Sports Clinic, Nagoya, JPN; 4 Department of Orthopedic Surgery, Tanaken Spine and Ophthalmology Clinic, Ichinomiya, JPN

**Keywords:** conservative treatment, dynamic ultrasound, far-lateral lumbar disc herniation, neural gliding, psoas major muscle, spinal nerve

## Abstract

Far-lateral lumbar disc herniation is a relatively uncommon condition for which conservative treatment is generally prioritized; however, patients often present with severe radicular pain or motor deficits. This report describes the case of a woman in her 50s with a left L4/5 far-lateral disc herniation, who presented with persistent anterior thigh pain. Although magnetic resonance imaging (MRI) clearly demonstrated the herniated fragment, dynamic ultrasonography revealed reduced flexibility and impaired gliding among the herniated fragment, the psoas major muscle, and the L4 spinal nerve. Based on these findings, manual and targeted therapeutic exercises were performed on the affected regions. Following these interventions, the patient’s anterior thigh pain during walking resolved, and neurological findings associated with L4 radiculopathy, including muscle weakness, sensory disturbance, and abnormal deep tendon reflexes, improved. A follow-up MRI at six months showed no reduction in the size of the herniation; however, no symptom recurrence was observed for up to one year. This case suggests that clinical improvement may occur even without morphological changes in the herniated disc, potentially through functional restoration of the nerve and surrounding tissues. Dynamic ultrasonography appears useful for identifying functional impairments and guiding treatment.

## Introduction

Lumbar disc herniation is a major cause of lower-limb pain and neurological deficits. Far-lateral disc herniation, which accounts for approximately 7-12% of all lumbar disc herniations, is relatively uncommon and most frequently occurs at the L3/L4 and L4/L5 levels [1]. In far-lateral herniations, the exiting nerve root or dorsal root ganglion is compressed outside the intervertebral foramen, often resulting in severe radicular pain and motor weakness. However, in the absence of marked neurological deficits, such as motor paralysis, conservative treatment is generally recommended, similar to the management of typical lumbar disc herniation [1,2]. Following the inflammatory response around the nucleus pulposus in disc herniation, scar formation and fibrosis may occur, leading to adhesion between the nerve and surrounding tissues, and a subsequent reduction in neural mobility [3-5]. Kobayashi et al. demonstrated during intraoperative straight leg raising (SLR) testing that adhesions between the nerve root and dura mater can restrict neural gliding and reduce blood flow, both of which improve after adhesion removal [6]. These findings suggest that neurological symptoms may result not only from morphological compression, but also from dynamic functional impairments due to restricted neural gliding. Magnetic resonance imaging (MRI) is useful for assessing morphological changes; however, its ability to evaluate the flexibility and mobility of nerves and adjacent structures is limited. In contrast, ultrasonography is minimally invasive and allows real-time visualization of the dynamic behaviors of nerves and muscles. To the best of our knowledge, no previous report has clearly documented ultrasonographic visualization of a far-lateral herniated fragment together with dynamic assessment of the psoas major muscle and L4 spinal nerve in such cases. This report presents a case in which conservative therapy guided by dynamic ultrasonographic assessment resulted in symptomatic improvement.

## Case presentation

Patient information

The patient was a woman in her 50s who worked as a cook. She presented to our clinic three weeks after the onset of left lower limb pain that developed without any identifiable trigger. MRI confirmed the diagnosis of a left L4/5 far-lateral lumbar disc herniation. The patient chose to undergo conservative treatment rather than surgery. During the initial visit, she reported severe pain in the anterior aspect of the left thigh (numerical rating scale (NRS), 10/10). She was able to walk approximately 100 m with the assistance of a cane and could not maintain a standing position for more than 5 min, making it impossible for her to continue working. The Oswestry Disability Index (ODI), one of the primary condition-specific outcome measures used in spine disorder management, ranges from 0% to 100%, with higher scores indicating greater disability [7]. An ODI score greater than 12% is generally considered indicative of functional disability [8]. At baseline, her ODI score was 78%, indicating marked impairment in the activities of daily living. Neurological examination revealed weakness in the left quadriceps femoris, iliopsoas, and tibialis anterior muscles, each graded 4/5 on the Manual Muscle Test (MMT). Sensory examination across the L2-S2 dermatomes showed normal bilateral findings, except for the left L4 distribution. The patient reported decreased sensation in the medial aspect of the left lower leg, which was first assessed using light touch and subsequently confirmed with pinprick testing. The left L4 patellar tendon reflex was absent (grade 0), whereas the right patellar tendon reflex was normal (grade 2). Reflexes were graded on a 0-4 scale, with 0 indicating absent (areflexia), 1 diminished (hyporeflexia), 2 normal, 3 brisk, and 4 very brisk (hyperreflexia). The femoral nerve stretch test was positive, reproducing anterior thigh pain with the patient in the prone position during knee flexion combined with hip extension [9]. In addition, hip range of motion was limited, with extension of -5°, flexion of 70°, adduction of 10°, and abduction of 30°.

Initial conservative treatment

During the first eight weeks after pain onset, conservative management focused on exercise therapy aimed at improving hip joint flexibility to reduce mechanical stress on the L4/5 intervertebral disc. Specifically, the program included active hip abduction-adduction exercises, gluteus maximus activation exercises, and stretching of the hip musculature. These interventions included improving thoracic mobility and providing postural education. Under the direction of the attending physician, celecoxib and pregabalin (25 mg/day) were prescribed for four weeks for pain relief, and the patient was instructed to wear a lumbar corset. No nerve root blocks were performed. Consequently, her pain gradually decreased, and at eight weeks, her ODI score improved from 78% to 42%. Hip range of motion improved to 90° of flexion, 20° of adduction, and 40° of abduction. Pharmacological therapy and the corset were discontinued at this time; however, hip extension remained limited to 5°, which reproduced anterior thigh pain, and anterior thigh pain during walking (NRS 6/10) persisted. In addition, vertical compression applied from the ventral side to the deep layer of the psoas major reproduced anterior thigh pain. Therefore, ultrasound-based re-evaluation and additional intervention were performed.

Re-evaluation and intervention using ultrasound

**Figure 1:**
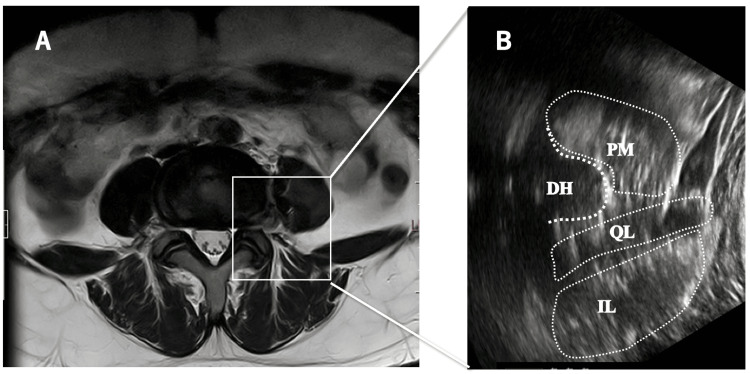
Magnetic resonance imaging (MRI) and short-axis ultrasonographic images. A: Axial MRI at the L4/5 level. B: Short-axis ultrasonographic image showing the psoas major muscle anterior to the herniated disc. PM, psoas major muscle; DH, disc herniation; QL, quadratus lumborum; IL, iliocostalis.

With the patient in the right lateral decubitus position, a short-axis “shamrock view” was obtained at the L4 level. The psoas major muscle, located anterior to the herniated fragment, was visualized by tilting the transducer anteriorly (Figure 1).

**Figure 2:**
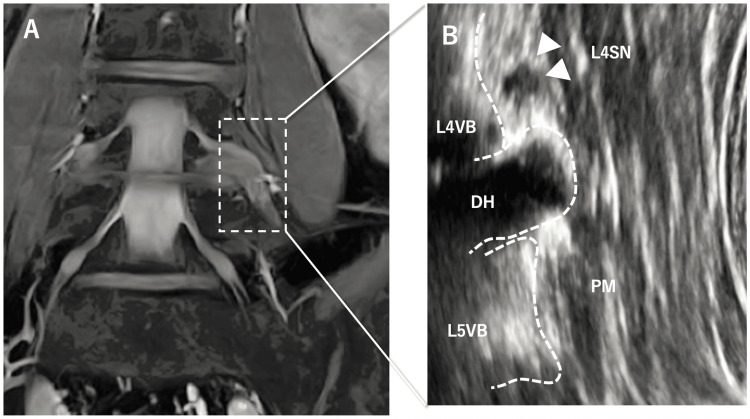
Magnetic resonance imaging (MRI) of the herniated disc and long-axis ultrasonographic images. A: Coronal MRI of the lumbar spine. B: Long-axis ultrasonographic image showing the L4 spinal nerve cranial to the herniated disc. L4VB, L4 vertebral body; L5VB, L5 vertebral body; L4SN, L4 spinal nerve (white arrow); DH, disc herniation; PM, psoas major muscle.

A longitudinal view of the psoas major muscle was subsequently obtained, allowing the identification of the L4 vertebral body, herniated disc fragment, and hypoechoic L4 spinal nerve (Figure 2). Under ultrasound guidance, vertical compression was applied laterally at the L4 vertebral level through the psoas major muscle toward the L4 spinal nerve. This maneuver reproduced the patient’s anterior thigh pain. Furthermore, during passive 5° hip extension, the superficial fibers of the psoas major muscle glided caudally.

However, the deeper tissues located beneath them, particularly the deep psoas fibers surrounding the herniated fragment (indicated by white arrows), showed minimal caudal displacement (Video 1).

**Video 1:** Dynamic ultrasonographic evaluation during passive hip extension before treatment. With passive hip extension, the superficial psoas major muscle demonstrated caudal gliding; however, there was minimal caudal movement of the deep portion of the psoas major muscle and perineural tissues surrounding the herniated disc material. L4VB, L4 vertebral body; L5VB, L5 vertebral body; L4SN, L4 spinal nerve (white arrow); DH, disc herniation; PM, psoas major muscle.

Based on these findings, an intervention was performed involving manual compression techniques. The compression technique was conducted for a total duration of 5 min. Manual vertical compression was applied to the psoas major muscle belly immediately adjacent to the disc herniation. The compression technique involved a continuous cycle of applying pressure for 5 s, followed by a 2-s release. The force applied was determined by achieving deformation of the deep layer of the psoas major muscle, as visualized on the ultrasound image. The objective of compression was to promote a reduction in localized tenderness without provoking radicular symptoms (Video 2).

**Video 2:** Manual compression technique and corresponding ultrasonographic imaging. A. Manual compression procedure: With the patient in the side-lying position, gentle manual compression was applied to the deep psoas major muscle and L4 spinal nerves. Note: In this case, the individual shown in the video is not the patient. B. Ultrasonographic findings during manual compression: Repeated vertical compression toward the L4VB was applied until tenderness decreased, with the aim of improving tissue flexibility around the L4 spinal nerve. The black arrow indicates the direction of manual compression. L4VB: L4 vertebral body; L5VB, L5 vertebral body; L4SN, L4 spinal nerve (white arrow); DH, disc herniation; PM, psoas major muscle.

As a result, caudal gliding of the deep tissues (white arrows) during hip extension improved, and the reproduced radiating pain upon L4 nerve compression disappeared (Video 3). One week after the intervention, the patient’s walking pain completely resolved (NRS 0/10), and the ODI improved to 0%. Muscle strength of the left quadriceps, iliopsoas, and tibialis anterior recovered to MMT 5/5, sensory deficits normalized to the level of the contralateral limb, and the patellar tendon reflex improved to a normal grade of 2. In addition, the femoral nerve stretch test result was negative, and a course of therapeutic exercises was concluded.

**Video 3:** Dynamic ultrasonographic evaluation of passive hip extension after treatment. Passive hip extension was performed in the same manner as before treatment, and improvement was observed in the caudal movement of the deep portion of the psoas major muscle and tissues surrounding the herniated disc material. L4VB, L4 vertebral body; L5VB, L5 vertebral body; L4SN, L4 spinal nerve (white arrow); DH, disc herniation; PM, psoas major muscle.

Clinical course

A follow-up MRI performed six months after symptom onset showed no reduction in the size of the herniated disc; however, the patient remained completely symptom-free, and no recurrence was observed at the one-year follow-up. Physical examination revealed an ODI score of 0% and an NRS score of 0, indicating the absence of pain or functional impairment. Quadriceps strength was maintained at 5/5, and no sensory deficits or abnormalities in the patellar tendon reflex were noted. Dynamic ultrasound assessment demonstrated that gliding between the L4 spinal nerve and the psoas major muscle remained well preserved, with no evidence of recurrent adhesion or impaired mobility following the resolution of inflammation (Table 1).

**Table 1:** Overview of clinical findings and therapeutic course. NRS, Numerical Rating Scale; ODI, Oswestry Disability Index; MMT, Manual Muscle Testing; FNST, Femoral Nerve Stretching Test; US, ultrasonography; N/A, not assessed; +, positive finding; -, negative finding.

Parameter	Initial visit (3 weeks after onset)	Week 8	Week 8 (immediately after intervention)	Week 9	6 months	1 year
Pain (NRS :0-10)	10	6	2	0	0	0
ODI (%)	78	42	N/A	0	0	0
Muscle strength (MMT)	4/5	4/5	5/5	5/5	5/5	5/5
L4 sensory deficit	+	+	-	-	-	-
Patellar tendon reflex	0	1	2	2	2	2
FNST	+	+	-	-	-	-
Imaging findings (MRI)	L4/5 lateral-type lumbar disc herniation	N/A	N/A	N/A	No reduction in herniation size	No reduction in herniation size
Treatment/intervention	Initiated conservative treatment (celecoxib, pregabalin 25 mg/day, lumbar orthosis), manual hip intervention	Re-evaluated with ultrasonography (medication discontinued), manual hip intervention	Ultrasound-guided manual intervention, manual hip intervention	Completion of exercise therapy	None	None

## Discussion

Previous reports have indicated that conservative treatment for lumbar disc herniation typically requires 10 to 52 weeks for symptomatic improvement. Although physical therapy and traction may reduce activity limitations and low back pain, their effects on radicular leg pain are generally limited [10-12]. In the present case, pain and limitations in daily activities improved within nine weeks, and the radicular symptoms in the lower limb resolved completely. This represents a relatively shorter recovery period and more favorable clinical outcome compared with typical timelines reported in the literature. As the L4 spinal nerve penetrates the psoas major muscle [13], lateral-type disc herniation places the herniated mass, nerve root, and psoas muscles in close proximity. Following inflammation, adhesions may occur between the nerve and muscle, leading to contracture and impaired neural gliding [3-5]. Although neural mobilization has been shown to improve symptoms and neural excursion more effectively than stretching [14], no previous report has identified the exact site of impaired neural gliding and targeted it for treatment. In this case, dynamic ultrasonography enabled the identification of gliding impairment between the L4 nerve and the psoas major muscle. Manual therapy directed at the adhesion site subsequently improved the dynamic interaction between the nerve and the psoas major, reducing traction stress on the nerve. As a result, anterior thigh pain rapidly decreased immediately after treatment, with substantial improvement in gait-related pain. This immediate response supports the possibility that functional factors, rather than structural changes alone, contributed substantially to symptom generation. Adhesion-related restriction of neural mobility is known to contribute to reduced nerve blood flow and ectopic firing [6,15]. In the present case, improved neural gliding likely alleviated these mechanisms, leading to pain reduction. Furthermore, the improvement in neural gliding was accompanied by the resolution of neurological symptoms, including nerve-related reductions in muscle output.

Recent advances in imaging quality and device miniaturization have enabled the widespread clinical use of ultrasound-guided peripheral nerve blocks, and the ability to identify spinal nerves on ultrasonography has been well-documented [16,17]. Specifically, spinal nerves are typically visualized as slightly hypoechoic, rounded structures surrounded by hyperechoic fat [17]. Furthermore, evaluating the gliding function with dynamic ultrasonography may contribute to assessing the potential reversibility of neural impairment prior to surgery, thereby aiding in surgical decision-making.

This study has several limitations. As this was a single-case report, the generalizability of the findings is limited, and the reproducibility of ultrasound-based gliding assessments requires further validation. In addition, serial MRI follow-up was insufficient. Future studies involving a larger number of cases are needed to clarify the frequency of this condition and to further evaluate the effectiveness of ultrasound-guided intervention. Another important consideration is that earlier recognition and intervention for gliding impairment may have enabled more rapid clinical improvement, underscoring the potential importance of early intervention.

## Conclusions

This case demonstrates that intervention based on dynamic ultrasonographic assessment is effective in patients with lateral-type lumbar disc herniation. Dynamic ultrasound provides crucial information on neural gliding that cannot be obtained from MRI, allowing for a clearer identification of therapeutic targets during conservative management. Furthermore, the evaluation of neural mobility may serve as a useful indicator of neural reversibility prior to surgery and may support clinical decision-making regarding surgical candidacy.
